# Draft genome of *Chloroflexu*s sp. MS-CIW-1, of the *Chloroflexus* sp. MS-G group from Mushroom Spring, Yellowstone National Park

**DOI:** 10.1128/mra.00710-23

**Published:** 2024-02-01

**Authors:** Amanda N. Shelton, Feiqiao B. Yu, Freddy Bunbury, Jia Yan, Carlos Rivas, Arthur Grossman, Devaki Bhaya

**Affiliations:** 1 Department of Plant Biology, Division of Biosphere Sciences and Engineering, Carnegie Institution for Science, Stanford, California, USA; 2 Chan Zuckerberg Biohub, Stanford, California, USA; SUNY College of Environmental Science and Forestry, USA

**Keywords:** *Chloroflexus*, phototrophic mat, chloroflexota, hot spring, anoxygenic phototroph, phototrophs, genomes, Mushroom Spring

## Abstract

*Chloroflexus* sp. MS-CIW-1 was isolated from a phototrophic mat in Mushroom Spring, an alkaline hot spring in Yellowstone National Park, WY, USA. We report the draft genome of 4.8 Mb consisting of 6 contigs with 3755 protein-coding genes and a GC content of 54.45%.

## ANNOUNCEMENT

*Chloroflexus* sp. MS-CIW-1 was isolated from a mat core sampled at 60°C in the runoff channels of Mushroom Spring, an alkaline hot spring in Yellowstone National Park, WY, USA (44.538714,–110.798022) on 2006/09/12. A liquid enrichment culture in DH10 medium (1) with 500 mg/L yeast extract and tryptone was serially diluted and then plated onto PE medium (2) at 50°C under continuous white light (50 µmol m^−2^ s^−1^). The edges of orange motile colonies were picked and passaged twice more in liquid and on plates. Axenicity was confirmed by light microscopy and by genome sequencing. This isolate is routinely used in our lab to study the physiology of filamentous anoxygenic phototrophs and their interactions with other members of the mat community. Cells are maintained in liquid PE medium without shaking. Our original analysis of the partial 16S sequence using the BLAST webserver (3) suggested the closest two 16S sequences were from *Chloroflexus* sp. Y-396-1 (GCF_000516515), from the nearby Octopus Spring, and *Chloroflexus* sp. MS-G (GCF_000735195), also from Mushroom Spring (4).

We isolated DNA by a phenol-chloroform-based method following bead beating (5). Illumina libraries were created with the Nextera XT Kit and were sequenced using a 2 × 150 bp configuration on Illumina’s iSeq, yielding 5,094,597 reads. For long-read sequencing, DNA was not sheared prior to library preparation with Oxford Nanopore’s SQK-LSK109 PCR-Free Ligation Kit, and sequencing was performed with the Oxford Nanopore MinION flow cell (FLO-MIN106) to obtain 391,464 reads with a median length of 7,000 bp. Basecalling was performed with Guppy. Read trimming and filtering were performed by BBTools (v38.73) (6), quality assessed with FastQC (v0.11.9) (7), followed by hybrid assembly using Unicycler (v0.4.8) (8), and quality control by QualiMap (v.2.2.2) (9) and QUAST (v5.0.2) (10) using the workflow as described by Jin et al. (11). The high-quality draft genome consists of 6 contigs, with an N50 of 3,270,211 bp, with a total length of 4,838,670 bp. Mean coverage was 209× for the short reads and 621× for long reads. The GC content was 54.45%.

We compared this genome to the available *Chloroflexus* genomes in RefSeq and found the closest relative by ANI [fastANIv0.1.3 on KBase (12, 13)] was strain MS-G with an identity of 98.4%. An alternative approach using GTDB-Tk [v1.7.0 on KBase (14, 15)] placed MS-CIW-1 in “s__Chloroflexus sp000735195,” the MS-G species group. All five genomes in this species group on GTDB R207 (16, 17) are MAGs or isolates from Yellowstone National Park. Two of the three 16S loci in MS-CIW-1 are identical, the third is 99.73% identical. These 16S are 99.52%–99.66% identical to MS-G (KR230107) and 98.06%–98.27% identical to Y-396-1 (AJ308498) [GeneiousPrime v2022.1.1 and MUSCLE v3.8.425 (18)]. MS-CIW-1 is the most contiguous genome available for the *Chloroflexus* sp. MS-G group.

We annotated MS-CIW-1 using the NCBI prokaryotic genome annotation pipeline (PGAP v6.5) (19), predicting 3,755 protein-coding genes (3,778 including pseudogenes), 3 16S rRNA loci, 50 tRNA, and 4 CRISPR arrays. The CRISPR systems were classified as type IIIA and type I by CRISPRCasfinder (accessed 06 July 2023) (20, 21). Pangenome analysis [KBase Compute Pangenome v0.0.7 (13)] with MS-G found 3,399 homolog families are shared between MS-G and MS-CIW-1. Genome comparison using Mauve aligner (22) on GeneiousPrime reveals different placements of some transposons and nearby genes between the two genomes, suggesting active transposition since these genomes split.

## Data Availability

This Whole-Genome Shotgun project has been deposited in DDBJ/ENA/GenBank under the accession no. JAUBWR000000000. The version described in this paper is version JAUBWR010000000. Reads have been deposited in SRA under accessions SRR25023911 and SRR25024071. All project data are available under the BioProject accession number PRJNA985735.
